# Objective Assessment of the Nature and Extent of Children’s Internet-Based World: Protocol for the Kids Online Aotearoa Study

**DOI:** 10.2196/39017

**Published:** 2022-10-11

**Authors:** Marcus Gurtner, Moira Smith, Ryan Gage, Anna Howey-Brown, Xinyi Wang, Tevita Latavao, Jeremiah D Deng, Sander P Zwanenburg, James Stanley, Louise Signal

**Affiliations:** 1 Department of Public Health University of Otago Wellington New Zealand; 2 Department of Information Science University of Otago Dunedin New Zealand

**Keywords:** public health, child health, internet, policy, methods, child, youth, methodology, student, human-computer interaction, digital health, eHealth, technology use, computer use, user experience, perception, attitude, internet use, screen time, perceived behavior, phone use, social media

## Abstract

**Background:**

Children under 18 years of age account for approximately 1 in 3 internet users worldwide. Largely unregulated, the internet-based world is evolving rapidly and becoming increasingly intrusive. There is a dearth of objective research globally on children’s real-time experiences of the internet-based world.

**Objective:**

This paper reports an objective methodology to study the nature and extent of children’s internet-based world, their engagement with it, and how this impacts their health and well-being.

**Methods:**

A total of 180 year 8 students from 12 schools will be recruited into the study within the Wellington region of Aotearoa, New Zealand. Children use Zoom video teleconferencing software to record real-time, screen-shared internet-based content, for 4 consecutive days. Data on demographics, health and well-being, and attitudes and perceived behaviors in relation to the internet-based world are collected. Phone screen-time balances are retrieved. Data collection commenced in June 2021 and is anticipated to be completed in 2023.

**Results:**

Recordings show children exploring diverse web-based settings and content, including personalized content curated by algorithms on platforms such as TikTok, YouTube, and Instagram. Preliminary analysis shows that the data can be used to study a wide range of topics. Behavioral Observation Research Interaction Software is being used to manually code recordings. Artificial Intelligence techniques are also being applied, including hashtag extraction, optical character recognition, as well as object, pattern, speech, and lyric recognition.

**Conclusions:**

This novel methodology reveals the unique internet-based experiences of children. It is underpinned by a commitment to ensuring that their rights are protected. It seeks to provide concrete evidence on internet usage in this group and to facilitate appropriate political and societal action to effectively regulate the internet-based world to prevent harm to children.

**International Registered Report Identifier (IRRID):**

DERR1-10.2196/39017

## Introduction

Children and adolescents aged under 18 years account for approximately 1 in 3 internet users worldwide and access the internet at increasingly younger ages [1]. The largely unregulated internet-based world is evolving rapidly and becoming increasingly intrusive owing to constant connectivity to “always on” gaming, social media, and other applications [2,3]. Children primarily use smartphones to navigate the internet-based world [2]. Smartphones fuel a “bedroom culture” of childhood, characterized by low levels of supervision and a highly personalized, private experience [1]. The internet-based world affords children access to reliable information and connectivity with friends and family. Simultaneously, it increases their likelihood of encountering harmful or distressing web-based content and behavior, such as marketing for harmful products, violent or discriminatory material, cyberbullying, or sexual solicitation [2].

There is unequivocal evidence linking children’s high screen-time with adiposity, depressive symptoms, and an unhealthy diet [4-6], alongside emerging literature about a range of other harms from internet-based interaction, such as exposure to marketing for unhealthy products; bullying; racist, discriminatory, or hate speech material; sexual and violent content; and websites advocating unhealthy or dangerous behaviors such as self-harm, suicide, and anorexia [1,2]. Nonetheless, there is a dearth of objective research globally on children’s real-time experiences of the internet-based world [2].

To the best of our knowledge, existing evidence in this area has relied predominantly on self-report data or observations made in controlled experimental conditions, owing in part to the difficulty of capturing engagement with personalized internet-based content [7]. Existing research typically considers the internet’s effect on children in terms of the time spent on the internet, as opposed to the nature of the content encountered [2]. Evidence remains especially scarce for preteens [2]. A method capable of capturing internet-based experiences in real time is urgently needed. This paper reports an objective methodology to study the nature and extent of children’s internet-based world, their engagement with it, and how this may impact their health and well-being.

## Methods

### Study Design

Kids Online Aotearoa (Kids Online) is a cross-sectional observational study of children’s real-time experiences of the internet-based world; it is currently in the field and due for completion in 2023. Kids Online aims to recruit 180 year 8 students (aged 11-13 years) from 12 schools in the Wellington region of Aotearoa, New Zealand, using a stratified sample design (see below). Participants use Zoom video teleconferencing software [8] to record real-time, internet-based, screen-shared content for 4 consecutive days (Thursday to Sunday, inclusive). Data on demographics; health and well-being; attitudes and behaviors in relation to the internet-based world; and daily phone use (screen-time) data balances are also collected.

### Ethical Approval

Ethical approval was obtained from the University of Otago Human Ethics Committee (Health; 20/006) to study the nature and extent of children’s internet-based world, their engagement with it, and how it may impact their health and well-being.

### Pilot Study

In 2019, a feasibility study was undertaken with medical student participants, which demonstrated that Zoom recordings enabled the study of adults’ internet-based world [9]. Building on this, a pilot study was conducted with 5 preteens from one school in late 2020. A focus group with the children revealed that the study was well explained, easy to undertake, and enjoyable. Children were familiar with Zoom, or similar applications, from internet-based learning and communication during the COVID-19 pandemic. Termination of Zoom recordings when locking phones and an overcomplicated instruction booklet were the key issues that the children highlighted. Consequently, during the full study, children were asked to disable their phone locking function, an additional briefing was added, and a simplified, generic instruction sheet was developed. The study protocol was updated and is available on the internet [10].

### Sampling and Recruitment

Sampling and recruitment are being undertaken in 2 stages. All 93 schools in the Wellington region with year 8 students are eligible for selection. Sampling is stratified to provide equal explanatory power [11] by school decile (socioeconomic measures: low=1-5, high=6-10) and student ethnicity (Māori, Pacific, non-Māori, and non-Pacific), using nationwide enrollment data from the Ministry of Education.

In total, 12 schools were randomly selected from the resulting 6 strata, using probability-proportional-to-size sampling methods (such that schools with a larger proportion of year 8 students in a stratum have a higher probability of invitation). The sampling method allows for a school to be selected for multiple ethnicities. Year 8 children are then randomly selected from the class list.

The total sample size is limited by feasibility and budget. Data analysis for projects using these data will account for the complex sampling design using sampling weights, appropriate management of stratification variables, and clustering of children within schools [12]. As of July 2022, a total of 22 students from 3 schools have participated.

### Data Collection

#### Selection

**Figure 1:**
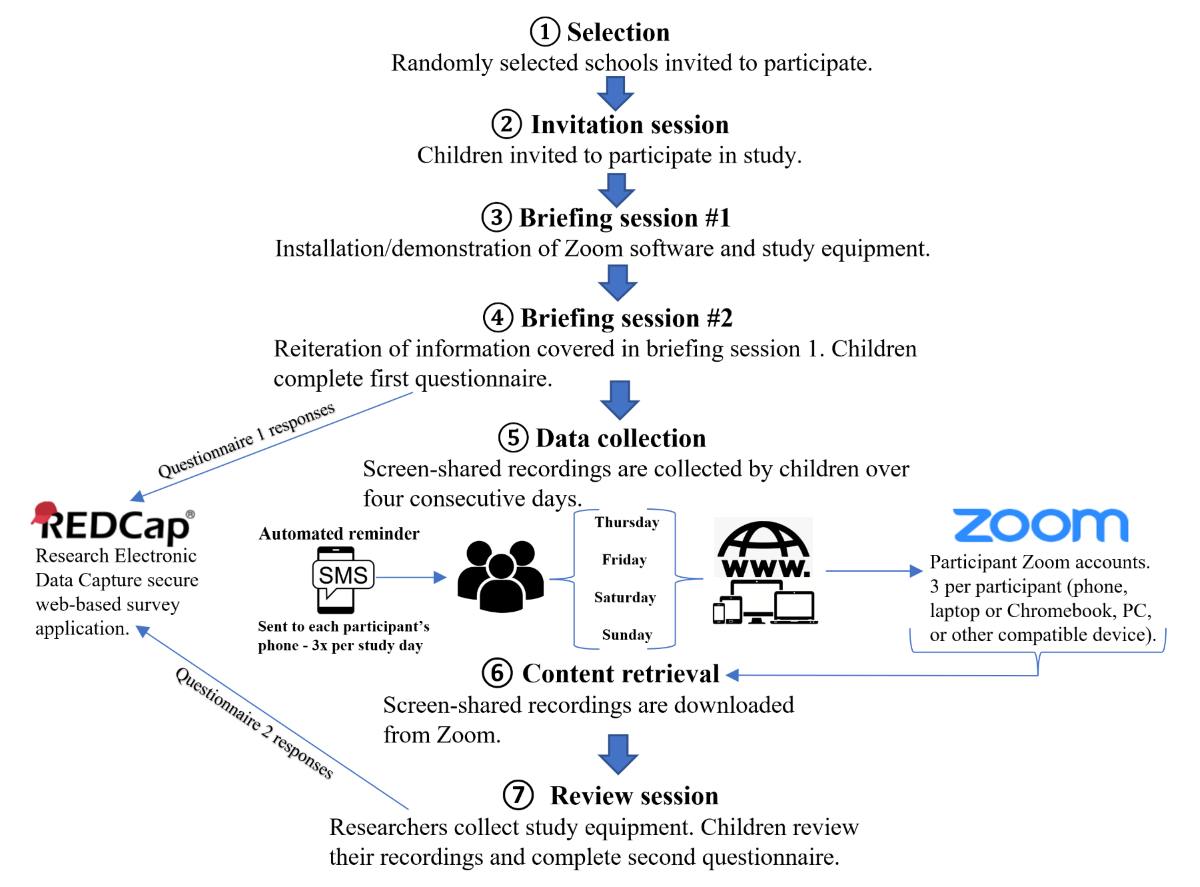
Participant recruitment and data collection.

Selected schools are invited to participate (Figure 1). School principals inform their school community about the study, and it proceeds if no objections are raised. Participating schools provide a list of eligible children by ethnicity. Children unable to undertake the study, owing to health or family circumstances, are excluded on the advice of senior staff. Eligible children for each ethnicity strata selected for that school are randomized using Excel (Microsoft Inc). They are invited to participate in a half-hour invitation session at their school. The number of invitees exceeds the number of required participants to reduce the burden on schools from multiple rounds of invitation. Written consent is obtained from participating schools, children’s parents or guardians, and children. Children are informed that their anonymity and that of third parties will be protected, and if any data are used in publications, all personally identifiable information will be obscured. The first 10 children on the list for each ethnic group who return signed consent forms are selected.

#### Briefings

Selected children are briefed twice about the study at school over 2 days, fitting around their timetables. Children are asked to bring their portable electronic devices. Researchers work with each child to ensure that Zoom is installed on their devices and teach them how to install it on other devices they use. Children are given 3 university-operated Zoom accounts to record up to 3 screen devices (mobile phones, laptops or Chromebooks, and PCs). Researchers text children their Zoom account log-in and password details. They are asked to log in to Zoom on each of their devices and to select the “stay signed in” button. Children are instructed to use Zoom to share their screen, internal device audio, and ambient sound captured via the device microphone any time they access the internet over the 4 days of the study. They are asked to use the internet as they normally would and to make a trial Zoom recording following the first briefing. The installation of Zoom and their ability to use it is confirmed at the second briefing.

Children are given the instruction sheet and a statement explaining the study to communicate to third parties if asked. An Alcatel LINKZONE wireless internet device is provided to participants. This enables internet access when other trusted networks are unavailable and prevents costs to participants through use of personal mobile data. Zoom recordings cause device batteries to discharge rapidly. To ensure that the children can charge devices without connecting to an electrical outlet, a Fast Charge power bank coupled with charging cables is also provided. Children are instructed to keep devices charged. An automated web-based SMS text messaging service (Vodafone multiTXT) is used to send 3 texts on each of the study days, reminding children to share their screen and to charge all devices. Researchers and children communicate via text as needed.

Children complete the first questionnaire on their attitudes toward and perceived behavior in relation to the internet-based world. The questionnaire is based on the Global Kids Online qualitative methodology for researchers [13]. A validated measure of self-reported general well-being is also included (the 5-item World Health Organization Well-Being Index) [14].

#### Content Retrieval

Children’s screen-shared recordings are downloaded from Zoom cloud storage on the Monday following data collection to a secure university server. The recorded content is subsequently deleted from Zoom. The researchers do not view the data at this stage.

#### Review

In the days following content retrieval, researchers conduct a review session with the children. They are reminded of the project’s commitment to maintain anonymity. Children are asked whether they recorded anything they did not wish the researchers to see. If so, that content is deleted by the researchers without being viewed. A second questionnaire is completed, which includes validated measures assessing anxiety (the 7-item Generalized Anxiety Disorder scale) [15], depression (the 9-item Patient Health Questionnaire) [16], and adolescent self-esteem (the Adolescent Self-esteem Questionnaire) [17]. Additional questions on sleep quality and habits, and device use prior to sleep are also included (see Multimedia Appendices 1 and 2 for the questionnaires). Participants’ height and weight are measured, and their feedback on the study is recorded. Wherever possible, children’s daily phone use (screen-time) balances are recorded for the week preceding and during the week of the study. This enables comparison of phone use prior to and during the study period.

### Quality Control

Researchers are trained in the study protocol. Data are collected by pairs of researchers, initially MG and LS. Other researchers assisted and took over once proficient. All wireless devices, charging cables, and battery packs are tested for functionality. Calibrated scales (Wedderburn HD-316) and stadiometers are used to calculate children’s weight and height to derive their BMI.

### Data Management

Data are downloaded to a secure university server and backed up to a password-protected external hard drive. Data access is restricted to members of the research team who sign a data release form, which includes strict data access protocols. Demographic information is anonymized using participant numbers.

### Data Analysis

Manual coding of recordings is conducted using the Behavioral Observation Research Interaction Software (version 7.11.1) [18], which permits events of interest to be coded using user-generated coding ethograms: “a catalogue or table of all the different kinds of behavior or activity observed" [19]. Artificial Intelligence (AI) pattern recognition techniques will also be used for data analysis.  First, various OpenCV [20] and Scikit-Image [21] utilities will be used to detect specific topics of interest—for example, TikTok or YouTube video scenes—using global and local template matching. Second, object detection will be carried out; for example, identifying fast food logos. It may be possible to detect multiple objects with one scan, further improving efficiency [22]. Third, Google Tesseract [23] will be used to analyze video frames and extract hashtags and other displayed words within a scene, allowing for lexicographic analysis and sentiment analysis. Further, machine learning techniques will be used to analyze the audio data, including speech and lyric recognition, and musical mood classification [24].

## Results

The Kids Online methodology enables objective investigation of the nature and extent of children’s internet-based world. The retrieved recordings depict children exploring diverse internet-based settings and content, including personalized content curated by algorithms on social media platforms such as TikTok, YouTube, and Instagram, the use of which is commonplace.

The method permits observation of; the complexity of the internet-based world, the speed at which transitions occur between settings, how children use technology to access and navigate the internet, and how this differs between home and school.

Characteristics of internet-based settings are clearly discernible, as are children’s interactions occurring within them, such as scrolling or browsing, clicking, posting, and communicating. On-screen events, such as pop-up advertisements, notifications, messaging, and alerts, are obvious and occur frequently.

As of July 2022, a total of 197 hours of real-time, screen-shared Zoom recordings have been uploaded by 22 children. This indicates that a substantial amount of data is likely to be retrieved. Participants reported that they recorded most of their internet use and had used the internet normally. Children cited forgetting to record as the main reason for data loss. Daily phone use (screen-time) balances were retrievable from most children’s phones. These permit comparisons of participant phone use (daily screen-time durations) preceding and during the study, approximating any effect due to the study itself. Additionally, the balances indicate data loss when compared to total duration for daily Zoom phone recordings.

The method permits a large sample size as participants collect data themselves, significantly reducing time and resources. To date, 1 in 4 children who were invited had participated, with no participant dropout or any expression of concern by third parties. No child has requested that any data be deleted. All children have reported that Zoom is easy to use. Zoom allows consistent uploading and archiving of intact recordings, unlike previous methods [25]. Conducting 2 briefing sessions ensures that participants can undertake the study correctly. Provision of mobile data enables consistent Zoom data collection, which is data-intensive and expensive, particularly in Aotearoa New Zealand.

**Figure 2:**
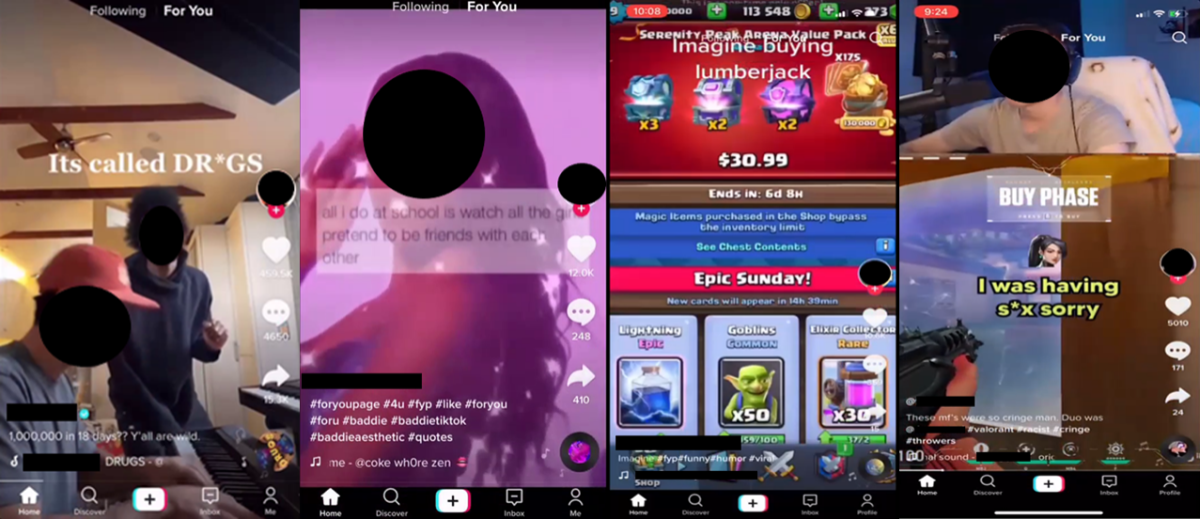
Examples of internet-based content encountered by children (left to right): a screenshot from TikTok on November 18, 2020, from video data captured by participant 4, depicting referencing to recreational drug use; a screenshot from TikTok on November 18, 2020, from video data captured by participant 4, depicting referencing to recreational drug use, profanity, and “bad girl” or risk-taking imagery; a screenshot from TikTok on September 25, 2021, from video data captured by participant 10, depicting in-game reward purchasing (requires real money to be used) or gambling-like content; and a screenshot from TikTok on September 25, 2021, from video data captured by participant 10, depicting gaming content. We received consent from all participants and their parents, caregivers, or other legal guardians to use their video data to generate screenshot images for use here. We created the images and have permission to use them.

**Figure 3:**
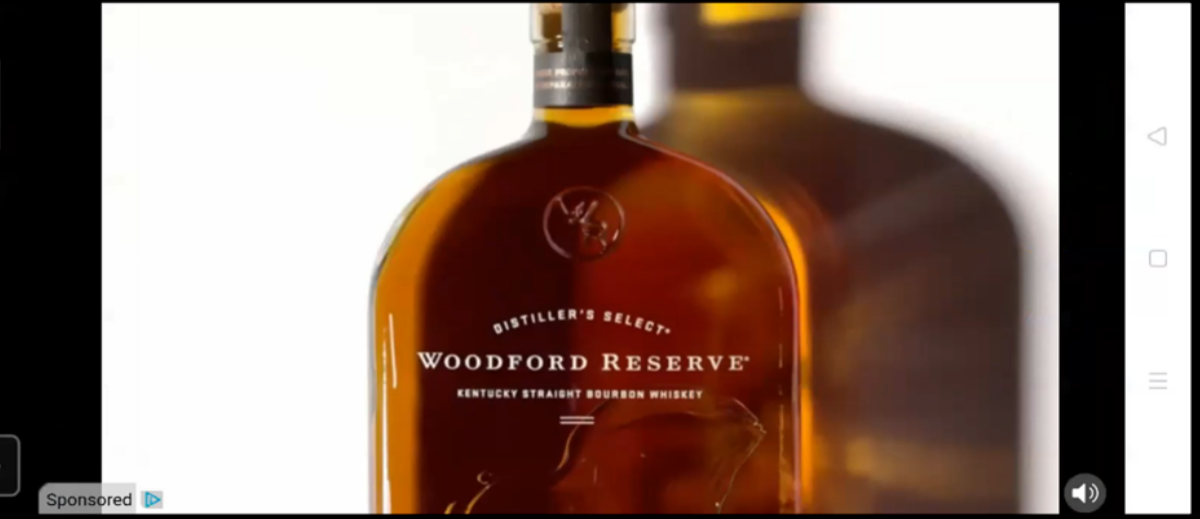
Screenshot taken from video data captured by participant 13 on September 23, 2021, depicting an in-game (mobile app) advertisement for Woodford Reserve Kentucky Straight Bourbon whiskey from Brown-Forman Corp. We received consent from all participants and their parents, caregivers, or other legal guardians to use their video data to generate screenshot images for use in publications. We created the images and have permission to use them.

**Figure 4:**
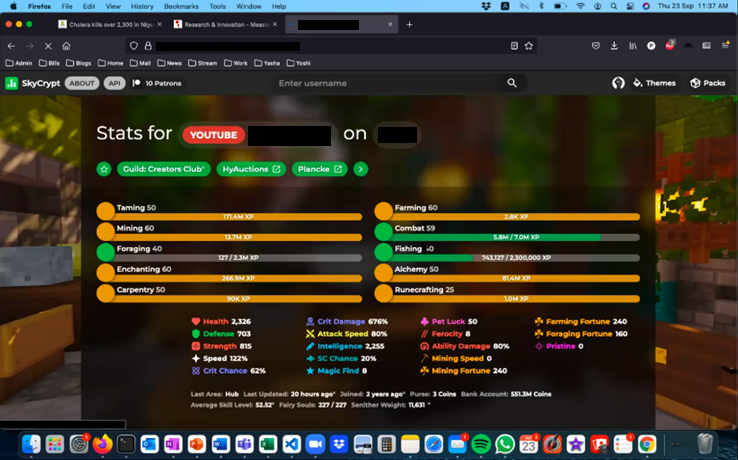
A screenshot recorded by participant 14 on September 23, 2021, depicting the complexity and type of internet-based content that may be seen by participants. This image shows a MacOS laptop being used in conjunction with the Mozilla Firefox web browser, depicting gaming-related content on the SkyCrypt website [26]. SkyCrypt is a free open-source stats viewer for Hypixel SkyBlock. The open tab text has been obscured because it contains potentially identifiable information. Multiple browser tabs are open and visible. Numerous applications are open as evidenced in the taskbar (icons at the bottom of the screen), owned by Apple, Dropbox, Mozilla, Spotify, Meta, Microsoft, Google, and others. We received consent from all participants and their parents, caregivers, or other legal guardians to use their video data to generate screenshot images for use in publications. We created the images and have permission to use them.

 Initial analysis suggests the data can be used to study a wide range of content (Figures 2-4), such as exposures to harmful advertising (Figure 3). Initial coding of data for gambling and gaming indicates that manual coding is possible, despite the complexity of the image data (Figures 2 and 4). Initial AI video analysis demonstrates that, for example, TikTok viewing sessions can be detected and extracted with 98% reliability.

## Discussion

### Expected Findings

The internet-based world affords children many benefits, such as connectivity, and access to educational and cultural content. Simultaneously, it increases their likelihood to encounter harm. Harms include marketing for unhealthy products; bullying; racist, discriminatory, or hate speech material; sexual and violent content; and websites advocating unhealthy or dangerous behaviors, such as self-harm, suicide, and anorexia.

The Kids Online methodology allows researchers to study the unique internet-based experiences of children and how these may impact their health and well-being. It enables children to document their internet-based world in real time via objective screen-share recordings. Retrieved recordings are highly personalized, their content is diverse, and they depict numerous web-based settings - providing researchers with individualized contexts and perspectives on internet usage among preteens, a demographic for which very little evidence currently exists. This insight cannot be garnered using researcher observation or survey research.

While only one-quarter of invited students have participated so far, this should still represent the broader experience of students in this age group. Researchers need to be technologically savvy given that children use a range of devices of different generations. It is also necessary to brief the children well, although they are often very technology literate. Manual coding of recordings is feasible, albeit time intensive. AI techniques will assist in reducing coding time.

Given that the wireless devices provided internet access that did not include restrictions that may be imposed by schools or parents, it is possible that participants used the internet more, or differently than normal. It is also possible that children selectively exclude or include certain internet-based content or behaviors while recording. However, children were instructed to use the internet as they normally would and largely reported doing so - comparison with phone use (screen-time) balances will provide some insight. Nevertheless, the data captured are a true record of the children's internet-based experiences.

Collectively, the retrieved recordings and demographic, attitude, and behavioral insights, and the health and well-being data provide a rich basis for future analyses of the nature and extent of all aspects of children’s internet-based world, their engagement with it, and the impact on their health and well-being. The method is legal, ethical, and acceptable to child participants, the adults in their lives, and those whom they interact with.

### Conclusions

The Kids Online Aotearoa methodology is a novel, objective approach to investigate children’s internet-based world. It aims to inform appropriate political and societal action to effectively regulate the internet-based world to ensure that the rights of children are upheld [27], thereby preventing web-based harm and enabling them to flourish.

## Appendix

Multimedia Appendix 1 Questionnaire 1 used in study.

Multimedia Appendix 2 Questionnaire 2 used in study.

## Abbreviations

AI: artificial intelligence
Kids Online: Kids Online Aotearoa

## Data Availability

The data analyzed during the current study will not be made publicly available, in accordance with our ethical agreement.
