# Supplementary material for: Objective Assessment of the Nature and Extent of Children’s Internet-Based World: Protocol for the Kids Online Aotearoa Study
Source: JMIR Res Protoc. 2022 Oct 11;11(10):e39017. doi: 10.2196/39017 (PMC9597417; doi:10.2196/39017)
Supplement: Multimedia Appendix 1 [file resprot_v11i10e39017_app1.pdf]

NZ Kids Online - Questionnaire #1

Hi :)

Remember, all of your answers will be kept private and your name will not be recorded.

Please take your time and answer as honestly as possible. By completing this questionnaire, you are contributing to pioneering research.

Thank you for making this possible!

Please complete all of the following questions.

Please enter the ID code that was assigned to you:

____
(Please take the time to enter this correctly. This code is used instead of your name to ensure that the answers you provide remain confidential)

1.) - Imagine that you are the Prime Minister for a day. This is your opportunity to make changes to the online world. What would you change about the online world, and why?

____
(Something you would change, and why you would change it.....)

2.) - Are you able to go online (use the internet) whenever you WANT to, while in the following locations?

(Make one selection per question)

	never	hardly ever	sometimes	often	very often	always
2.a) - At home?	○	○	○	○	○	○
2.b) - At school?	○	○	○	○	○	○
2.c) - Out in public, or while using public transport?	○	○	○	○	○	○

3.) - Are you able to go online (using the internet) whenever you NEED to, while in the following locations?

(Make one selection per question)

	Never	Hardly ever	Sometimes	Often	Very often	Always
3.a) - At home?	○	○	○	○	○	○
3.b) - At school?	○	○	○	○	○	○
3.c) - Out in public, or while using public transport?	○	○	○	○	○	○

4.a) - Which of the following devices do you have access to at home?

(it doesn't need to be a device you use, it just has to be one that is at your home)

- ☐ TV
- ☐ Desktop computer
- ☐ Laptop
- ☐ Tablet/iPad
- ☐ Mobile phone
- ☐ Gaming console
- ☐ Kindle/E-Reader
- ☐ Other(s)
- ☐ None

(Select all that apply)

What is/are the other device(s) you use?

____

(Please list here)

4.b) - Of those devices that you have access to at your home - which do you actually use to explore online?

TV	◯ Yes<br>◯ No
Desktop computer	◯ Yes<br>◯ No
Laptop	◯ Yes<br>◯ No
Tablet/iPad	◯ Yes<br>◯ No
Mobile phone	◯ Yes<br>◯ No
Gaming console	◯ Yes<br>◯ No
Kindle/E-reader	◯ Yes<br>◯ No
4.c) - You said that you have access to these 'other' devices at your house; [ac_dshome_other4a].<br><br>Which of them do you actually use to explore online?	__________________________________<br>(Please list here)
5.) - How frequently do you go online when you are at home?	◯ Almost all the time (I go online very often)<br>◯ More than once an hour<br>◯ At least once an hour<br>◯ Several times each day<br>◯ Daily or almost daily<br>◯ At least every week<br>◯ At least every month<br>◯ Once or twice a year<br>◯ Never<br>◯ Don't know<br>(Pick one - the most relevant to you)
6.a) - Which of the following devices do you have access to at school?<br><br>(it doesn't need to be a device you actually use, but one that is available to use if you needed or wanted to)	☐ TV<br>☐ Desktop computer<br>☐ Laptop<br>☐ Tablet/iPad<br>☐ Mobile phone<br>☐ Gaming console<br>☐ Kindle/E-Reader<br>☐ Other<br>☐ None<br>(Select all that apply)
What is/are the other device(s) you use?	__________________________________<br>(Please type here)

6.b) - Of those devices that you have access to at your school - which do you actually use to explore online?

TV	◯ Yes<br>◯ No
Desktop computer	◯ Yes<br>◯ No
Laptop	◯ Yes<br>◯ No
Tablet/iPad	◯ Yes<br>◯ No
Mobile phone	◯ Yes<br>◯ No
Gaming console	◯ Yes<br>◯ No
Kindle/E-reader	◯ Yes<br>◯ No
6.c) - You said that you have access to these 'other' devices at your school; [ac_dschool_oth6a].<br><br>Which of them do you actually use to explore online?	______________________<br>(Please list here)
7.) - How often do you go online when you are at school?	◯ Almost all the time (I go online very often)<br>◯ More than once an hour<br>◯ At least once an hour<br>◯ Several times each day<br>◯ Daily or almost daily<br>◯ At least every week<br>◯ At least every month<br>◯ Once or twice a year<br>◯ Never<br>◯ Don't know<br>(Pick one - the most relevant to you)
8.a) - What is your favourite device to use to go online?	◯ TV<br>◯ Desktop computer<br>◯ Laptop<br>◯ Tablet/iPad<br>◯ Mobile phone<br>◯ Gaming console<br>◯ Kindle/E-Reader<br>◯ Other<br>◯ Don't really mind/no preference<br>◯ Don't know<br>(Pick one)

What is the 'other' device that is your favourite?

____
(Please type here)

8.b) - Why is that device your favourite?

____
(A short answer is absolutely fine)

9.) - While online (using the internet), how often do you see the following:

(Make one selection per question)

	More than once daily	At least once every day	At least once every week	Once or twice a month	Once or twice a year	Never	Don't know
9a.) - Alcohol or alcohol marketing?	◯	◯	◯	◯	◯	◯	◯
9b.) - Gambling or marketing for gambling?	◯	◯	◯	◯	◯	◯	◯
9c.) - Junk food or marketing for junk food?	◯	◯	◯	◯	◯	◯	◯

10.) - Do your parents limit the amount of time you’re allowed to spend online on school days (Monday-Friday)?

◯ Every day
◯ Sometimes, somedays
◯ Never
◯ I don't know
(Pick one)

11.) - On a typical day during the school week (Monday-Friday), what is the total time you spend online (using the internet)........

(Make one selection per question)

	None	0-9 minutes	10-19 minutes	20-29 minutes	30-45 minutes	About an hour	1-2 hours	2-4 hours	4+ hours	Don't know
11a.) - While at home?	◯	◯	◯	◯	◯	◯	◯	◯	◯	◯
11b.) - While at school?	◯	◯	◯	◯	◯	◯	◯	◯	◯	◯
11c.) - While in public spaces (not including public transport)?	◯	◯	◯	◯	◯	◯	◯	◯	◯	◯
11d.) - While travelling in any type of vehicle (including public transport)?	◯	◯	◯	◯	◯	◯	◯	◯	◯	◯

12.) - Do your parents limit the amount of time you’re allowed to spend online on weekend days? (Saturdays and Sundays)?

◯ Yes - On Saturday but NOT Sunday
◯ Yes - On Sunday but NOT Saturday
◯ Yes - on BOTH Saturday & Sunday
◯ No - they don't limit the amount of time I spend online on either Saturday or Sunday
◯ I don't know
(Pick one - the most relevant to you)

13.) - On a typical weekend day (Saturday & Sunday), what is the total amount of time you spend online (using the internet).............

(Make one selection per question)

	None	0-9 minutes	10-19 minutes	20-29 minutes	30-45 minutes	About an hour	1-2 hours	2-4 hours	4+ hours	Don't know
13a.) - While at home?	○	○	○	○	○	○	○	○	○	○
13b.) - While in public spaces (not including public transport)?	○	○	○	○	○	○	○	○	○	○
13c.) - While travelling in any type of vehicle (including public transport)?	○	○	○	○	○	○	○	○	○	○

14.) - Do your parents have rules or limits about the amount of time you spend using screens for these activities?

(Make one selection per activity)

	Yes	No	Don't know
14.a) - Watching TV or DVDs	○	○	○
14.b) - Streaming video (Netflix/Prime Video/Lightbox/YouTube etc.)	○	○	○
14.c) - Gaming	○	○	○
14.d) - Texting/messaging using a mobile phone	○	○	○
14.e) - Browsing the internet	○	○	○
14.f) - Using social media websites/apps (Facebook, Instagram, Snapchat, TikTok etc.)	○	○	○
14.g) - Reading books (using a Kindle/other device)	○	○	○

15.) - Which of the following social media platforms do you use?

(Select all the ones you use)

- ☐ Facebook
- ☐ Facebook Messenger
- ☐ YouTube
- ☐ WhatsApp
- ☐ Instagram
- ☐ TikTok
- ☐ QQ
- ☐ Snapchat
- ☐ Pinterest
- ☐ Reddit
- ☐ Twitter
- ☐ LinkedIn
- ☐ Other
- ☐ None

(Select all the ones you use)

What is/are the 'other' platform(s) you use?

____
(Please list here)

16.) - Thinking about when you are exploring online, how important are the following to you?

(Make one selection per statement)

	Strongly agree	Somewhat agree	Neither agree nor disagree	Somewhat disagree	Strongly disagree
16.a) - Introducing me to new people	○	○	○	○	○
16.b) - Allowing me to connect and share with people like me	○	○	○	○	○
16.c) - Connecting me with support networks or services	○	○	○	○	○
16.d) - Letting me express myself	○	○	○	○	○
16.e) - Relaxing and having fun	○	○	○	○	○

17.) - Thinking about when you are exploring online, how important are the following to you?

(Make one selection per statement)

	Strongly agree	Somewhat agree	Neither agree nor disagree	Somewhat disagree	Strongly disagree
17.a) - Improving my education and learning	◯	◯	◯	◯	◯
17.b) - Keeping me up to date with news and current affairs	◯	◯	◯	◯	◯
17.c) - Teaching me about issues I care about	◯	◯	◯	◯	◯
17.d) - To take action on issues I care about	◯	◯	◯	◯	◯
17.e) - Teaching me about taking care of the environment	◯	◯	◯	◯	◯
17.f) - Providing access to health information	◯	◯	◯	◯	◯

18.) - Thinking about when you are exploring online, how important are the following to you?

(Make one selection per statement)

	Strongly agree	Somewhat agree	Neither agree nor disagree	Somewhat disagree	Strongly disagree
18.a) - To create content and share with others	◯	◯	◯	◯	◯
18.b) - Strengthening my cultural identity	◯	◯	◯	◯	◯
18.c) - Strengthening my religious identity	◯	◯	◯	◯	◯
18.d) - Strengthening my existing relationships with my family	◯	◯	◯	◯	◯
18.e) - Letting me contribute to decision-making in my community	◯	◯	◯	◯	◯
18.f) - To buy or sell things	◯	◯	◯	◯	◯
18.g) - Encouraging me to develop new skills	◯	◯	◯	◯	◯

19.) - Imagine that suddenly, you could no longer use any of your devices to go online........

(Make one selection per statement)

	Strongly agree	Somewhat agree	Neither agree nor disagree	Somewhat disagree	Strongly disagree
19.a) - I wouldn't be able to talk to my friends	◯	◯	◯	◯	◯
19.b) - I'd get bored, there is nothing else to do	◯	◯	◯	◯	◯
19.c) - People wouldn't like me as much	◯	◯	◯	◯	◯
19.d) - My friends and family go online a lot so I wouldn't fit in	◯	◯	◯	◯	◯
19.e) - I wouldn't be able to keep up to date with the world	◯	◯	◯	◯	◯
19.f) - I would be stressed or worried without my devices	◯	◯	◯	◯	◯
19.g) - I wouldn't be able to escape from the real world	◯	◯	◯	◯	◯

20.) - What do you spend most of your time doing when you're online?

◯ Education - school work/homework, other study
◯ Entertainment (not social media) - videos, tv shows, movies (Netflix, Lightbox etc), music (Spotify, Apple music etc)
◯ Browsing social media websites/applications
◯ General browsing
◯ Looking into things that interest you
◯ Buying/selling things
◯ Messaging/communicating
◯ Online gaming
◯ Other
(Pick one)

Please specify:

____
(Please type here)

21.) - Over the past two weeks............

(Make one selection per statement)

	All the time	Most of the time	More than half of the time	Less than half of the time	Some of the time	At no time
21.a) - I have felt cheerful and in good spirits	○	○	○	○	○	○
21.b) - I have felt calm and relaxed	○	○	○	○	○	○
21.c) - I have felt active and vigorous	○	○	○	○	○	○
21.d) - I woke up feeling fresh and rested	○	○	○	○	○	○
21.e) - My daily life has been filled with things that interest me	○	○	○	○	○	○
