# Supplementary material for: Objective Assessment of the Nature and Extent of Children’s Internet-Based World: Protocol for the Kids Online Aotearoa Study
Source: JMIR Res Protoc. 2022 Oct 11;11(10):e39017. doi: 10.2196/39017 (PMC9597417; doi:10.2196/39017)
Supplement: Multimedia Appendix 2 [file resprot_v11i10e39017_app2.pdf]

NZ Kids Online 2021 - Questionnaire 2

Hi :)

Remember, all of your answers will be kept private and your name will not be recorded.

Please take your time and answer as honestly as possible. By completing this questionnaire, you are contributing to pioneering research.

Thank you for making this possible!

Please complete all of the following questions.

Please enter the ID code that was assigned to you:

____
(Please take the time to enter this correctly. This code is used instead of your name to ensure that the answers you provide remain confidential.)

22. Over the last 2 weeks, how often have you been bothered by the following problems? (Make one selection per statement)

	Not at all	Several days	Over half the days	Nearly everyday
22.a) - Feeling nervous, anxious, or on edge	◯	◯	◯	◯
22.b) - Not being able to stop or control worrying	◯	◯	◯	◯
22.c) - Worrying too much about different things	◯	◯	◯	◯
22.d) - Trouble relaxing	◯	◯	◯	◯
22.e) - Being so restless that it's hard to sit still	◯	◯	◯	◯
22.f) - Becoming easily annoyed or irritable	◯	◯	◯	◯
22.g) - Feeling afraid as if something awful might happen	◯	◯	◯	◯

23.a) - What time do you usually wake up on a normal school day? (Monday – Friday)

- ◯ 5-5:29am
- ◯ 5:30-5:59am
- ◯ 6-6:29am
- ◯ 6:30-6:59am
- ◯ 7:00-7:29am
- ◯ 7:30-7:59am
- ◯ 8:00-8:29am
- ◯ 8:30-9am

(Pick one - the most relevant to you)

23.b) - Around what time do you normally go to bed.........but NOT to sleep - on a school night (Monday-Friday)?

- ◯ 6-6:59pm
- ◯ 7-7:29pm
- ◯ 7:30-7:59pm
- ◯ 8:00-8:29pm
- ◯ 8:30-8:59pm
- ◯ 9:00-9:29pm
- ◯ 9:30-9:59pm
- ◯ 10:00-10:29pm
- ◯ 10:30-10:59pm
- ◯ 11:00-11:29pm
- ◯ 11:30-11:59pm
- ◯ Midnight-1am
- ◯ 2am

(Pick one - the most relevant to you)

23.c) - Around what time do you usually fall asleep - on a normal school night (Monday-Friday)?

◯ 6-6:59pm
◯ 7-7:29pm
◯ 7:30-7:59pm
◯ 8:00-8:29pm
◯ 8:30-8:59pm
◯ 9:00-9:29pm
◯ 9:30-9:59pm
◯ 10:00-10:29pm
◯ 10:30-10:59pm
◯ 11:00-11:29pm
◯ 11:30-11:59pm
◯ Midnight-1am
◯ 2am
(Pick one - the most relevant to you)

24.) - Over the past 2 weeks, how often have you been bothered by any of the following problems? (Make one selection per statement)

	Not at all	Several days	More than half the days	Nearly everyday
24.a) - Having little interest or pleasure in doing things	○	○	○	○
24.b) - Feeling down, depressed, or hopeless	○	○	○	○
24.c) - Having trouble falling or staying asleep, or sleeping too much	○	○	○	○
24.d) - Feeling tired or having little energy	○	○	○	○
24.e) - Having a poor appetite or overeating	○	○	○	○
24.f) - Feeling bad about yourself - or that you are a failure or have let yourself or your family down	○	○	○	○
24.g) - Having trouble concentrating on things, such as reading the newspaper or watching television	○	○	○	○
24.h) - Moving or speaking so slowly that other people could have noticed? Or the opposite - being so fidgety or restless that you have been moving around a lot more than usual	○	○	○	○
24.i) - Having thoughts that you would be better off dead or of hurting yourself in some way	○	○	○	○

25.a) - Around what time do you usually wake up on a normal Saturday morning?

○ 5-5:29am
○ 5:30-5:59am
○ 6-6:29am
○ 6:30-6:59am
○ 7:00-7:29am
○ 7:30-7:59am
○ 8:00-8:29am
○ 8:30-9am
○ 9-9:29am
○ 9:30-9:59am
○ 10:00-10:29am
○ 10:30-10:59am
○ 11:00-11:29am
○ 11:30-11:59am
○ Noon-1pm
(Pick one - the most relevant to you)

25.b) - Around what time do you normally go to bed.........but NOT to sleep - on a Saturday night?

◯ 6-6:59pm
◯ 7-7:29pm
◯ 7:30-7:59pm
◯ 8:00-8:29pm
◯ 8:30-8:59pm
◯ 9:00-9:29pm
◯ 9:30-9:59pm
◯ 10:00-10:29pm
◯ 10:30-10:59pm
◯ 11:00-11:29pm
◯ 11:30-11:59pm
◯ Midnight-1am
◯ 2am
(Pick one - the most relevant to you)

25.c) - Around what time do you usually fall asleep - on a normal Saturday night?

◯ 6-6:59pm
◯ 7-7:29pm
◯ 7:30-7:59pm
◯ 8:00-8:29pm
◯ 8:30-8:59pm
◯ 9:00-9:29pm
◯ 9:30-9:59pm
◯ 10:00-10:29pm
◯ 10:30-10:59pm
◯ 11:00-11:29pm
◯ 11:30-11:59pm
◯ Midnight-1am
◯ 2am
(Pick one - the most relevant to you)

26.a) - What time do you usually wake up on a normal Sunday morning?

◯ 5-5:29am
◯ 5:30-5:59am
◯ 6-6:29am
◯ 6:30-6:59am
◯ 7:00-7:29am
◯ 7:30-7:59am
◯ 8:00-8:29am
◯ 8:30-9am
◯ 9-9:29am
◯ 9:30-9:59am
◯ 10:00-10:29am
◯ 10:30-10:59am
◯ 11:00-11:29am
◯ 11:30-11:59am
◯ Noon-1pm
(Pick one - the most relevant to you)

26.b) - Around what time do you normally go to bed.........but NOT to sleep - on a Sunday night?

◯ 6-6:59pm
◯ 7-7:29pm
◯ 7:30-7:59pm
◯ 8:00-8:29pm
◯ 8:30-8:59pm
◯ 9:00-9:29pm
◯ 9:30-9:59pm
◯ 10:00-10:29pm
◯ 10:30-10:59pm
◯ 11:00-11:29pm
◯ 11:30-11:59pm
◯ Midnight-1am
◯ 2am
(Pick one - the most relevant to you)

26.c) - Around what time do you usually fall asleep - on a normal Sunday night?	◯ 6-6:59pm<br>◯ 7-7:29pm<br>◯ 7:30-7:59pm<br>◯ 8:00-8:29pm<br>◯ 8:30-8:59pm<br>◯ 9:00-9:29pm<br>◯ 9:30-9:59pm<br>◯ 10:00-10:29pm<br>◯ 10:30-10:59pm<br>◯ 11:00-11:29pm<br>◯ 11:30-11:59pm<br>◯ Midnight-1am<br>◯ 2am<br>(Pick one - the most relevant to you)
27.a) - Before shutting your eyes and trying to go to sleep - when would you have typically, last used a screen device (such as; phone, laptop, TV..)?	◯ 2+ hours before trying to sleep<br>◯ 1 hour before trying to sleep<br>◯ 30 minutes before trying to sleep<br>◯ 10 minutes before trying to sleep<br>◯ Just before trying to sleep<br>◯ Don't know<br>(Pick one - the most relevant to you)
28.) - If you wake up during the night or before you need to get up in the morning, do you ever check your phone or another electronic device?	◯ Yes<br>◯ No<br>◯ Don't know<br>(Pick one)
28.a) - Which of the following most closely relates to you?	◯ I wake up every night (7 nights/week) and always check my phone or another electronic device before going back to sleep<br>◯ I wake up every night (7 nights/week) and sometimes check my phone or another electronic device before going back to sleep<br>◯ I wake up some nights (3-6 nights/week) and always check my phone or another electronic device before back to sleep<br>◯ I wake up some nights (3-6 nights/week) and sometimes check my phone or another electronic device before back to sleep<br>◯ I rarely wake up during the night, but if I do, I always check my phone or another electronic device before going back to sleep<br>◯ I rarely wake up during the night, but if I do, I sometimes check my phone or another electronic device before going back to sleep<br>(Pick one - the most relevant to you)

29.) - These questions ask you how you feel about yourself. Please answer each question. Remember there are no right or wrong answers, just answer honestly (Make one selection per statement).

	Almost all of the time	A lot of the time	Some of the time	A little of the time	Hardly ever
29.a) - I am able to stand up for myself and what I believe in	○	○	○	○	○
29.b) - How I feel about myself depends on what others think of me	○	○	○	○	○
29.c) - I feel I can be myself around other people	○	○	○	○	○
29.d) - I make an effort to look good	○	○	○	○	○
29.e) - Overall I feel good about my abilities compared to others (e.g. at school, playing sports or socially)	○	○	○	○	○
29.f) - If I make an innocent mistake I let it get me down	○	○	○	○	○
29.g) - I feel useless	○	○	○	○	○

30.) - These questions ask you how you feel about yourself. Please answer each question. Remember there is no right or wrong answer. Answer as honestly as possible (Make one selection per statement).

	Strongly agree	Agree	Neither agree nor disagree	Disagree	Strongly disagree
30.a) - Overall I like who I am	◯	◯	◯	◯	◯
30.b) - I am a good person who has a lot to offer	◯	◯	◯	◯	◯
30.c) - I feel that I am a valuable person who is at least equal to other people	◯	◯	◯	◯	◯
30.d) - How I feel about my body makes me feel less confident	◯	◯	◯	◯	◯
30.e) - I feel confident in my abilities to achieve the things I set my mind to	◯	◯	◯	◯	◯
30.f) - I think other people like me	◯	◯	◯	◯	◯

31.) - Imagine you are the Prime Minister for a day and had the opportunity to make changes to the online world.

What would you change about the online world, and why?

____
(Something you would change, and why you would change it.....)
